# Clinical endpoints in the controlled human challenge model for *Shigella*: A call for standardization and the development of a disease severity score

**DOI:** 10.1371/journal.pone.0194325

**Published:** 2018-03-28

**Authors:** Chad K. Porter, Amanda Lynen, Mark S. Riddle, Kawsar Talaat, David Sack, Ramiro L. Gutiérrez, Robin McKenzie, Barbara DeNearing, Brittany Feijoo, Robert W. Kaminski, David N. Taylor, Beth D. Kirkpatrick, A. Louis Bourgeois

**Affiliations:** 1 Enteric Disease Department, Infectious Disease Directorate, Naval Medical Research Center, Silver Spring, MD, United States of America; 2 Bloomberg School of Public Health, Johns Hopkins University, Baltimore, MD, United States of America; 3 School of Medicine, Johns Hopkins University, Baltimore, MD, United States of America; 4 Department of Enteric Infections, Walter Reed Army Institute of Research, Silver Spring, MD, United States of America; 5 Drug Development Global Program, PATH, Seattle, WA, United States of America; 6 University of Vermont College of Medicine, Vaccine Testing Center, Department of Medicine, Burlington, VT, United States of America; 7 Enteric Vaccine Initiative, PATH, Washington, DC, United States of America; New York State Department of Health, UNITED STATES

## Abstract

**Background:**

Since 1946 the controlled human infection model (CHIM) for *Shigella* has been used to improve understanding of disease pathogenesis, describe clinical and immunologic responses to infection and as a tool for vaccine development. As the frequency and intent for use in vaccine comparisons increases, standardization of the primary endpoint definition is necessary.

**Methods:**

Subject-level data were obtained from previously conducted experimental *Shigella* CHIM studies. Signs and symptoms severity were categorized consistently across all studies. Sign and symptom correlations were estimated and univariate models were utilized to describe the association between stool output and other *Shigella*-attributable signs and symptoms. Multiple correspondence and hierarchical clustering analyses were performed to describe the co-occurrence of signs and symptoms. A disease score is proposed based on the co-occurrence of these events.

**Results:**

Data were obtained on 54 subjects receiving 800 to 2000 colony forming units (cfu) of *S*. *flexneri*. The median maximum 24 hour stool output was 514 ml (IQR: 300, 998 ml) with a median frequency of 6 (IQR: 4, 9). Subjects reported abdominal pain or cramps (81.5%), headache (66.7%) and anorexia (64.8%), 50.0% had a fever and 27.8% had gross blood in multiple loose stools. Multiple correspondence analyses highlighted co-occurrence of symptoms based on severity. A 3-parameter disease severity score predicted shigellosis endpoints and better differentiated disease spectrum.

**Conclusion:**

Dichotomous endpoints for *Shigella* CHIM fail to fully account for disease variability. An ordinal disease score characterizing the breadth of disease severity may enable a better characterization of shigellosis and can decrease sample size requirements. Furthermore, the disease severity score may be a useful tool for portfolio management by enabling prioritization across vaccine candidates with comparable efficacy estimates using dichotomous endpoints.

## Introduction

*Shigella* species are a leading cause of inflammatory diarrheal disease in endemic regions, and is especially problematic in children living in low-middle income countries, in refugees, travelers, and deployed military personnel [1–4]. *Shigella* are spread through fecal-oral transmission such as via contaminated food and water, and person to person contact, and has a low infective dose making it particularly challenging in settings of poor sanitation and overcrowding [5–7]. Globally, among children less than five years of age, mortality estimates approximate 54,900 deaths annually (95% uncertainty intervals: 27,000–94,700), accounting for 50% of the diarrhea-attributed deaths in this population [8]. In addition, *Shigella* are likely one of several pathogens that contribute to long-term adverse health outcomes including growth faltering [1, 9].

Diarrhea and dysentery have long been known as major medical problems during military campaigns, but it was not until the post-World War II era that the impact of travelers’ diarrhea was clearly delineated. Studies in the 1950s and 1960s identified diarrhea as the most common cause of illness for travelers to less-developed countries [10]. The importance of *Shigella* in military operations has been repeatedly described. As therapeutic options narrow, due to increasing antibiotic resistance, the need for a safe and effective *Shigella* vaccine becomes more pressing [11]. An expert panel convened by the Child Health and Nutrition Research Initiative of the World Bank identified *Shigella* as one of the highest priorities for long-term vaccine development [12]. The Product Development for Vaccines Advisory Committee (PDVAC) of the WHO has also recently endorsed the continued importance of *Shigella* vaccine development [13, 14]. Furthermore, the Department of Defense has issued directives (DoD Directive 6205.3; BUMEDINSTR 5450.171) for the development of vaccines against bacillary causes of diarrhea including *Shigella*.

The *Shigella* controlled human infection model (CHIM) has been used since 1946 to facilitate understanding of host-pathogen interaction, assess immunologic responses and to evaluate the efficacy of prophylactic prototype vaccine candidates (Table 1). At present the model has been successfully used in many academic institutions including, but not limited to, University of Maryland, Johns Hopkins University, United States Army Medical Research Institute for Infectious Diseases, University of Cincinnati, and Baylor University. We recently published a systematic review of the *Shigella* human challenge model to analyze study-specific factors and their associations with clinical outcomes [7]. As part of that review it was noticeable that no standardized definition of shigellosis had been widely adopted. Additionally, it was clear that dichotomous endpoints may not be sufficiently granular to differentiate some of the more significant manifestations observed. To that end, we sought to better characterize the disease profile of shigellosis observed in the CHIM and to develop a disease severity scoring algorithm for *Shigella*-attributable clinical illness, similar to what has recently been developed and applied for enterotoxigenic *Escherichia coli* CHIM studies [15]. This scale could then be externally validated in subsequent *Shigella* CHIM trials with the ultimate goal being validated outcome measures for use in future clinical trials that would improve comparability of results across trials.

**Table 1:** Uses of the *Shigella* CHIM to assess the efficacy of investigational products or prior challenge.

Product type	Product	Naïve attack rate (n/N)	Treated attack rate (n/n)	Efficacy (%)	Reference
Homologous rechallenge	2457T	22/39	3/15	64	DuPont ‘72 [23]
“	2457T	11/12	3/11	71	Kotloff ‘95 [24]
“	53G	8/12	0/6	100	Herrington ‘90 [25]
Antibiotic prophylaxis	Rifaximin	6/15	0/15	100	Taylor ‘06 [26]
Passive antibodies	Bovine colostral Ig	5/11	0/10	100	Tacket ‘92 [27]
Vaccine	Heat-Killed whole cell	19/30	18/25	-14	Shaughnessy ‘46 [28]
“	Irradiated whole cell	19/30	23/28	-30	Shaughnessy ‘46 [28]
“	EcSF2a^1^	6/24, 52/88	1/15, 30/68	73, 25	DuPont ‘72 [23]
“	Streptomycin-dependent mutant^1^	6/24, 52/88	3/31, 16/53	61, 49	DuPont ‘72 [23]
“	SC602	6/7	0/7	100	Coster ‘99 [29]
“	EcSF2a-2	12/14	10/16	27	Kotloff ‘95 [18]
“	Proteosome	13/13	9/14	36	Durbin ‘01 [30]
“	IVP_NAT_	8/12	7/10	-5	Harro ‘09 [31]
“	WRSS1^2^	5/10	3/10	40	Pitisuttithum ‘16 [32]
“	Flexyn2a	18/29	13/30	30	Talaat ‘17 [19]

1. Studies performed with challenge doses of 180 cfu and 10^4^ cfu; attack rates and efficacy estimates presented for both challenge doses in ascending order.

2. Study performed in Thai adults yielded lower than anticipated naïve attack rates

## Methods

For this study, individual subject data across selected studies (Table 2) were pooled for analysis. This analysis of pooled individual data affords the opportunity for more detailed analyses while avoiding some of the potential biases inherent in analyzing summary statistics of all study participants as is commonly conducted in systematic reviews [16]. Studies were selected based on the availability of individual subject-level files and consistency in recording clinical endpoints.

**Table 2:** Studies utilized to characterize *Shigella* CHIM endpoints and to develop a disease severity score.

Reference	PI	N	Dose (cfu)
NCT00485134	Harro	22	800
Antimicrob Agents and Chemother. 2008;52(3). [33]	Taylor	15	1500
Infect Immun. 1999; 67(7) [29]	Coster	7	2000
Clin Infec Dis 2006; 42(9) [26]	Taylor	10	1500

This study was limited to experimental infection studies in which subjects received a defined dose of a strain of *S*. *flexneri* and the investigative team provided individual subject-level data. Eligible studies included trials to develop an experimental challenge model, to characterize strain pathogenicity and immunogenicity or to evaluation of preliminary efficacy of a vaccine candidate (naïve subjects and unprotected vaccinees).

This study is an expansion of prior efforts to summarize human *Shigella* challenge studies [7]. As such, the data from the studies summarized as part of that research project have been abstracted (with 100% verification) into Microsoft® Excel. No subject-identifiable information was included in data abstractions.

A multiple correspondence analysis (MCA) was conducted to assess how symptoms correspond across subjects participating in *Shigella* CHIM studies. MCA is an analytical method utilized to detect and display the underlying structure of a set of nominal categorical data utilizing Euclidean distances [17]. MCA also allows graphically data display to describe the relationship between numerous nominal and/or ordinal data. Data are converted to a K by K table of all pairwise tabulations and when graphed on a two dimensional graph, the more proximal the variables, the more similar their distribution. This analysis was utilized to observe and describe clusters of signs and symptoms useful in the development of the clinical scoring algorithm. Additionally, descriptive analyses were conducted to describe the subset of clinical signs and symptoms and stool output across study characteristics. Based on the distribution of these signs, symptoms and diarrhea characteristics, ordinal approaches were explored in an effort to develop a disease severity scoring scale.

Three separate scores were developed as follows 1) clinical signs, 2) subject-reported symptoms, and 3) stool output. The goal was to combine the three scores into a single disease severity parameter. For clinical signs and symptoms, data from the MCA were utilized to identify relevant ‘clusters’ of symptom severity. These symptom clusters were assessed and amalgamated to ensure equal distribution across an ordinal spectrum of illness. This illness spectrum was assessed across inocula doses and diarrhea severity (based on per-protocol definitions). Similarly, the distribution of stool output was assessed using statistical cut-points such as median and interquartile ranges (IQR) to establish an ordinal range of stool output observed from prior experimental infections. This ordinal range was explored across inocula and diarrhea severity. These scores were combined to develop a composite, ordinal disease severity score. The ability of the score to predict dichotomous endpoints as well as hierarchical clusters of subjects based on the distribution and severity of clinical signs and symptoms post-challenge was assessed using logistic (binomial or ordinal) regression.

Hierarchical cluster analysis was used to create subject-based clusters based on the severity of self-reported symptoms and measured objective signs and stool output. These clusters were utilized to internally validate the developed disease severity score by being assessed as an independent variable in an ordinal logistic regression model with each subject’s disease score used as the dependent variable. Subjects were also dichotomized using previously applied endpoints as follows:

1. diarrhea (≥2 loose stools ≥200 grams over 48 hours or a single loose stool ≥300 grams) OR fever (oral temperature ≥100.0°F) OR gross blood confirmed by hemoccult in ≥1 loose stool [18]
2. severe diarrhea (≥6 loose stools in 24 hours or >800 grams of loose stool in 24 hours) OR moderate diarrhea (4–5 loose stools in 24 hours or 401–800 grams of loose stool in 24 hours) AND [fever (oral temperature >100.4°F) or with moderate-severe enteric/constitutional symptoms (nausea, vomiting, abdominal cramps/pain, myalgia, arthralgia, rigors, tenesmus, fecal urgency)] OR gross blood confirmed by hemoccult in ≥2 loose stools in 24 hours and enteric/constitutional symptoms (nausea, vomiting, abdominal cramps/pain, myalgia, arthralgia, rigors, tenesmus, fecal urgency) [19].

The study protocol was reviewed and approved by the Institutional Review Boards at the Naval Medical Research Center and the Johns Hopkins School of Public Health in compliance with all applicable Federal regulations governing the protection of human subjects and a waiver of informed consent. All statistical analyses were performed in SAS version 9.3 (SAS Institute, Cary, NC) and JMP 12.0.

## Results

**Fig 1:**
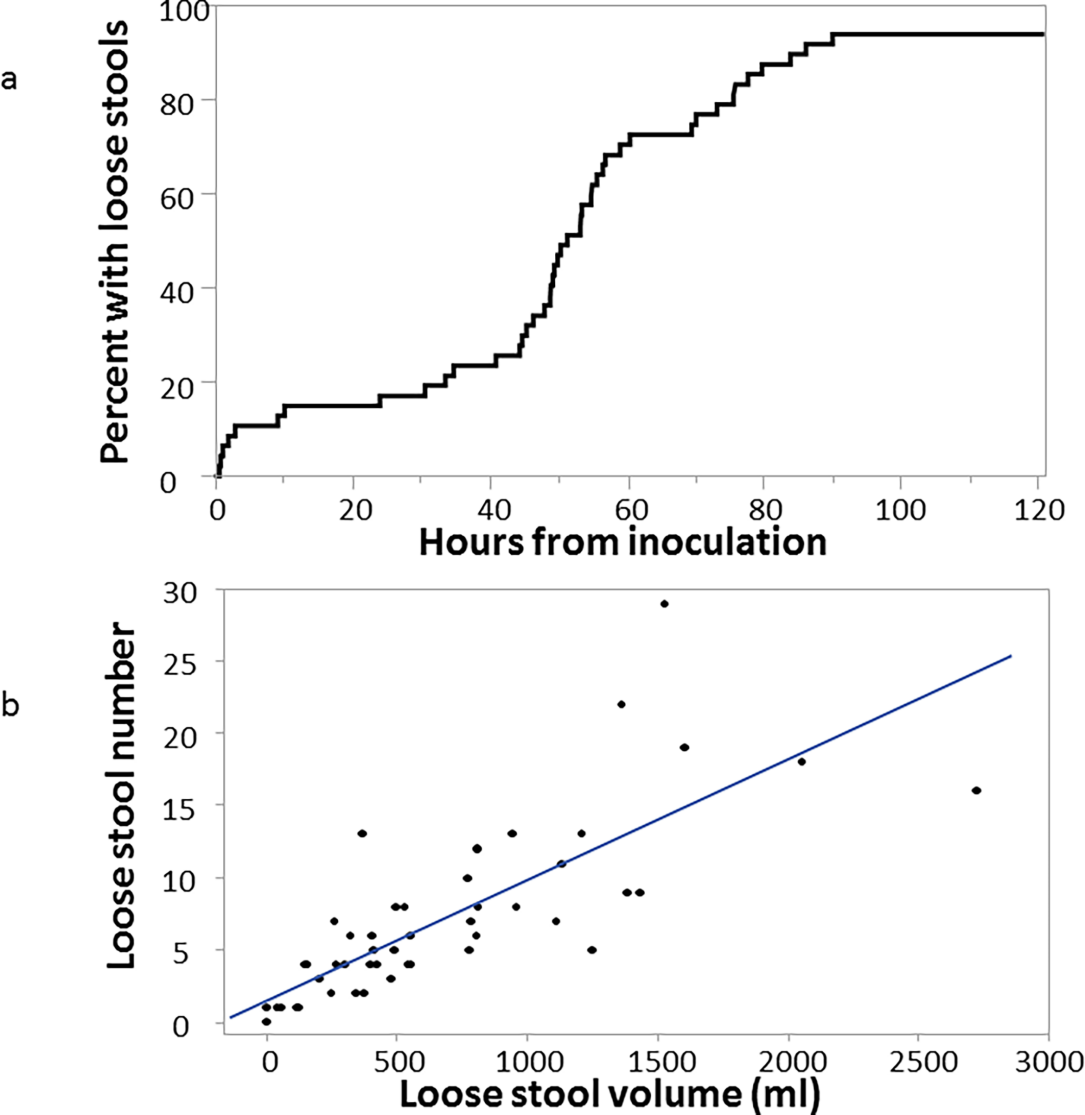
Stool output information in four CHIMs with *S*. *flexneri* 2a strain 2457T. 1A. Time to first loose stool from time of inoculation. The line indicates the proportion of subjects with at least 1 loose stool over time (in hours) following inoculation with *S*. *flexneri* 2a strain 2457T. 1B. Correlation between maximum number and volume of loose stools in any 24 hour period post-inoculation. Each dot represents each subject’s maximum 24 hour loose stool output by frequency and volume. The line represents the linear correlation between the maximum 24 hour frequency and volume.

Data were abstracted and collated on a total of 54 subjects with a distribution of signs and symptoms as shown in Table 3. The majority (75.9%) of subjects had diarrhea, most characterized as ‘moderate’ or ‘severe’. Abdominal pain or cramps were also common with 59.3% characterizing the symptom as moderate to severe intensity (ie, interfering or preventing their normal activities). Severe malaise was also quite common, reported in 42.6% of the subjects. The presence of gross blood in multiple loose stools was observed in approximately a quarter (27.8%) of subjects. Other signs and symptoms, including fever, nausea, vomiting, myalgia, arthralgia and anorexia were reported. Following challenge the majority of subjects began passing loose or unformed stools at approximately 50 hrs (Fig 1A). The maximum number and volume of loose stools in any 24 hour post challenge period showed a strong correlation (Pearson’s ρ = 0.88; p<0.001) (Fig 1B) with a median output of 5 loose stools (IQR: 2, 8) totaling a median of 417 ml (IQR: 155, 847 ml).

**Table 3:** Frequency (percent) of *Shigella*-attributed signs and symptoms in four CHIMs with *S*. *flexneri* 2a strain 2457T.

	Mild	Moderate	Severe	Any
Diarrhea^1^	7.4	24.1	44.4	75.9
Fever^2^	9.3	11.1	29.6	50.0
Nausea^3^	13.0	16.7	16.7	46.3
Abdominal pain/cramps^3^	22.2	20.4	38.9	81.5
Malaise^3^	10.6	4.3	42.6	57.4
Headache^3^	22.2	14.8	9.3	66.7
Myalgia^3^	13.0	11.1	18.5	42.6
Arthralgia^3^	3.7	9.3	7.4	20.4
Anorexia^3^	25.9	9.3	29.6	64.8
Vomiting				24.1
Dysentery^4^				27.8

1. mild, 2–3 loose stools in 24 hrs totaling <400 ml; moderate, 4–5 loose stools in 24 hrs or 401–800 ml loose stool in 24 hrs; severe, ≥6 loose stools in 24 hrs or > 800 ml loose stool in 24 hrs

2. mild, >100.4°F; moderate, >101.1°F; severe, >102.1°F

3. mild, does not interfere with normal activities; moderate, interferes with normal activities; severe, prevents normal activities

4. gross blood in multiple loose stools confirmed by hemoccult

**Fig 2:**
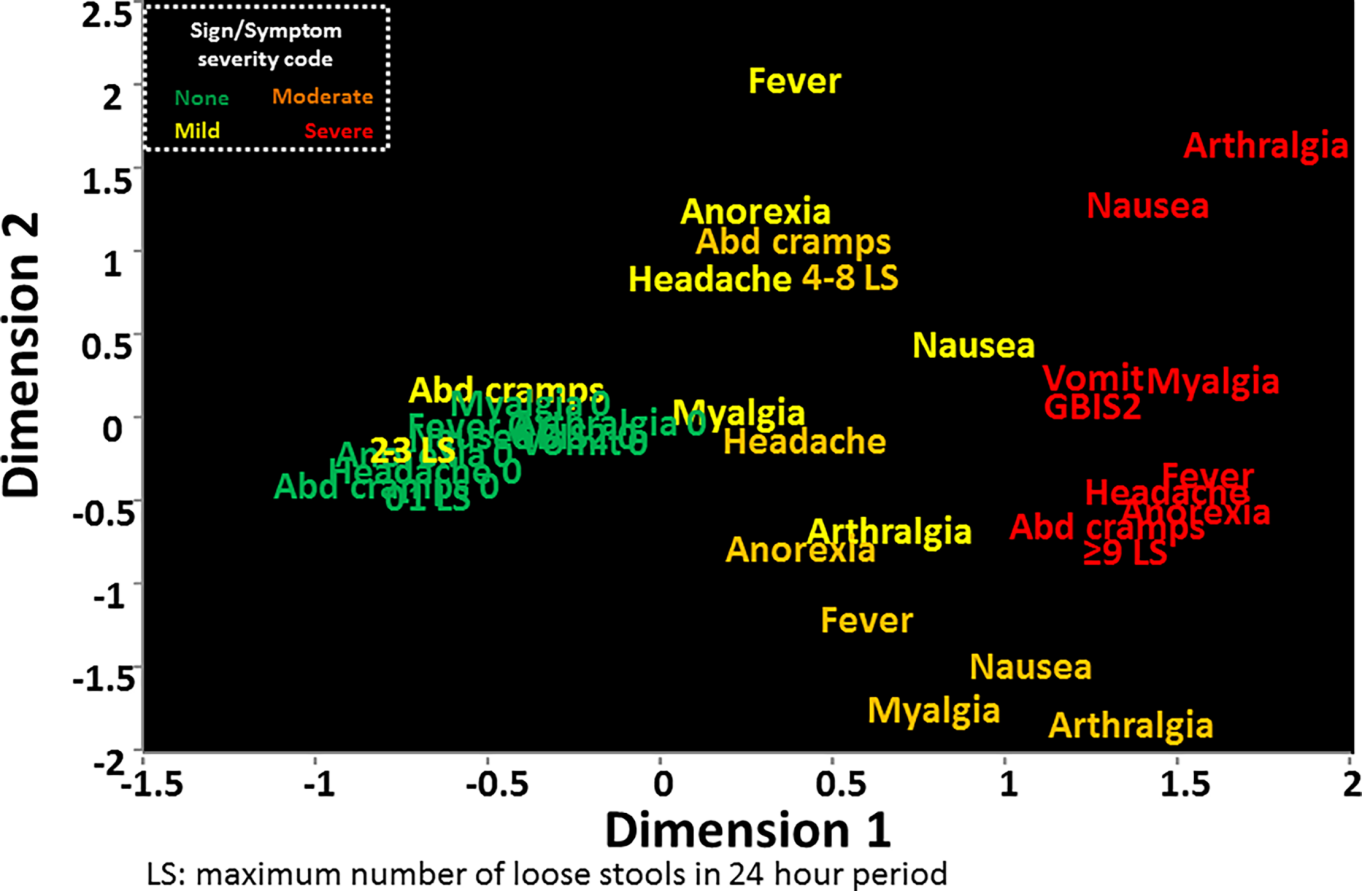
Multiple correspondence analysis of signs and symptoms observed during four CHIMs with *S*. *flexneri* 2a strain 2457T.

Multiple correspondence analysis of the signs and symptoms of shigellosis as well as the maximum number of loose stools in 24 hours (based on quartiles of output) in the CHIM is shown in Fig 2. Across dimensions 1 and 2, severe signs and symptoms (coded in red) appeared to co-occur. Moderate signs and symptoms (coded in orange) were interspersed with mild and severe symptoms. In particular, moderate arthralgia, nausea and myalgia appeared to correspond more with severe signs and symptoms while moderate anorexia, fever, headache and abdominal cramps corresponded more with mild signs and symptoms (coded in yellow). The lack of signs and symptoms appeared to occur concurrently, though often with the presence of mild abdominal cramps.

**Fig 3:**
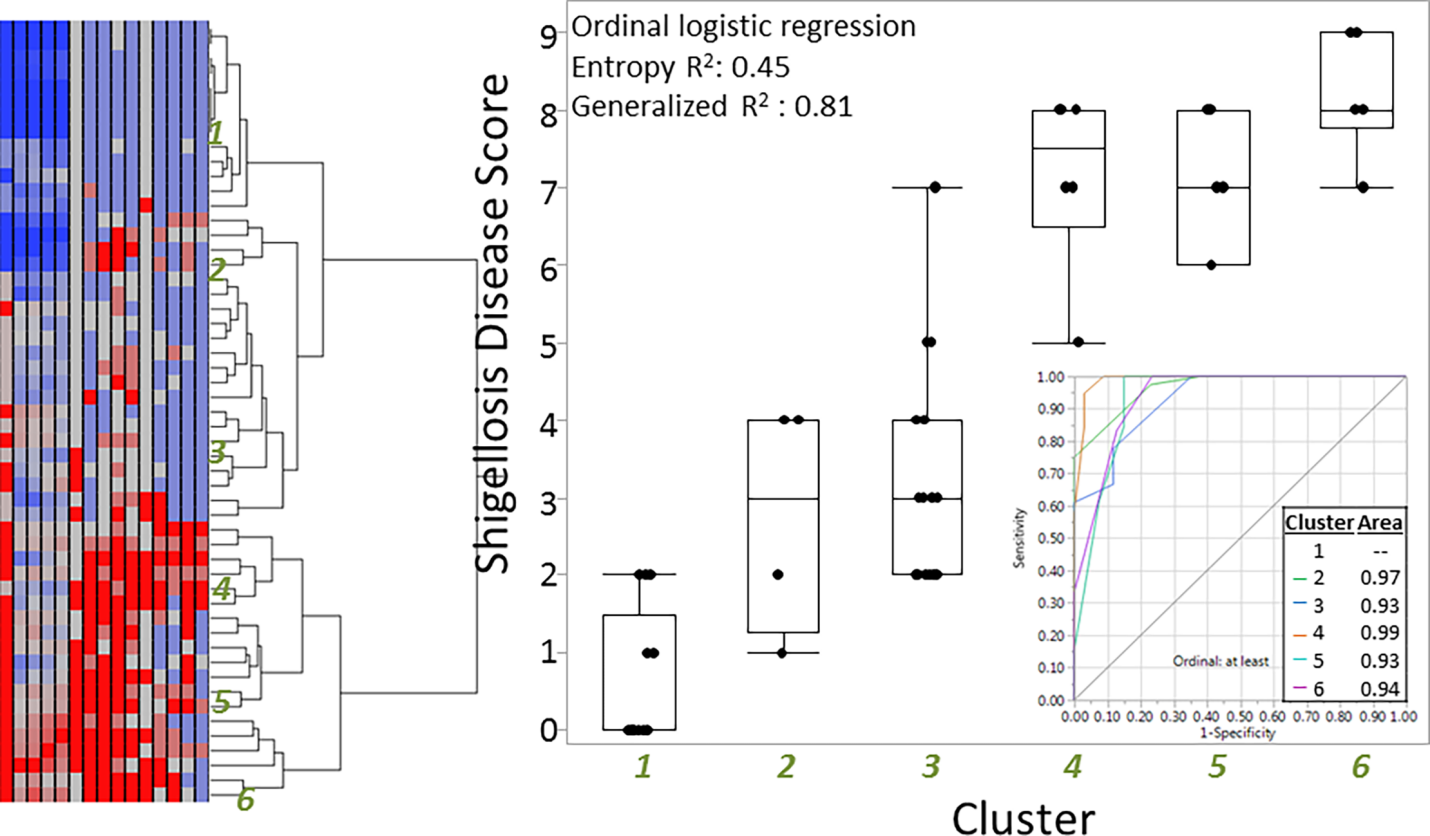
Results of hierarchical cluster analysis of subject signs and symptoms and correlation with developed *Shigella* CHIM disease severity score. Footnote: Signs and symptoms were used to identify clusters of disease profiles (numbered 1–6). Those clusters were then assessed as an ordinal predictor of the *Shigella* CHIM disease score. Box and whisker plots are utilized to demonstrate the distribution of the disease score across each sign/symptom cluster. The receiver operator curves demonstrate the ability of the disease score to predict disease cluster.

Based on the distribution of signs and symptoms as well as the parameters routinely collected as part of the *Shigella* CHIM, we developed a three parameter disease score (Table 4) based on self-reported symptoms, clinical signs and stool output with the goals of parsimony, approximations to normality, and optimization of predicting relevant binomial endpoints. The development of the specific score is described below. The score was applied iteratively to the 54 subjects to ensure optimal classification based on each subject’s disease profile. We internally validated the disease score by developing hierarchical clusters of subjects based on their signs and symptoms (Fig 3). As shown in the box and whisker plot, the median disease score increased as the prevalence and severity of shigellosis signs and symptoms also increased. This resulted in areas under the receiver-operator curve (ROC) >0.9 for all cluster levels. We also performed a principal component analysis (PCA), utilizing the signs and symptoms proposed (S1 Fig) and showed a strong, statistically significant correlation between component 1 and the disease score (S2 Fig). Additionally, we assessed the disease score across the dose ranges tested and identified a clear, statistically significant positive correlation (Spearman’s ρ = 0.38; p = 0.005) between dose and disease score with median scores of 2 (IQR: 0, 5), 4 (2, 7.5) and 8 (7, 8) at doses of 800, 1500 and 2000 colony forming units, respectively.

**Table 4:** Proposed *Shigella* CHIM disease severity score.

Parameter	Outcome	Score
**Objective signs**	Gross blood in ≥2 loose stools (hemoccult confirmed)	1
	Maximum temperature (°F); >101.1	1
	Vomiting	1
**Subjective symptoms**	More than one of the following as severe: arthralgia, nausea, myalgia, headache, anorexia, abd cramps/pain	3
	Any one of the following as severe: arthralgia, nausea, myalgia, headache, anorexia, abd cramps/pain	2
	More than one of the following as moderate: arthralgia, nausea, myalgia, headache, anorexia, abd cramps/pain	1
**Loose stool output** **(max 24 hr freq)**	0–1	0
	2–3	1
	4–8	2
	≥9	3

**Fig 4:**
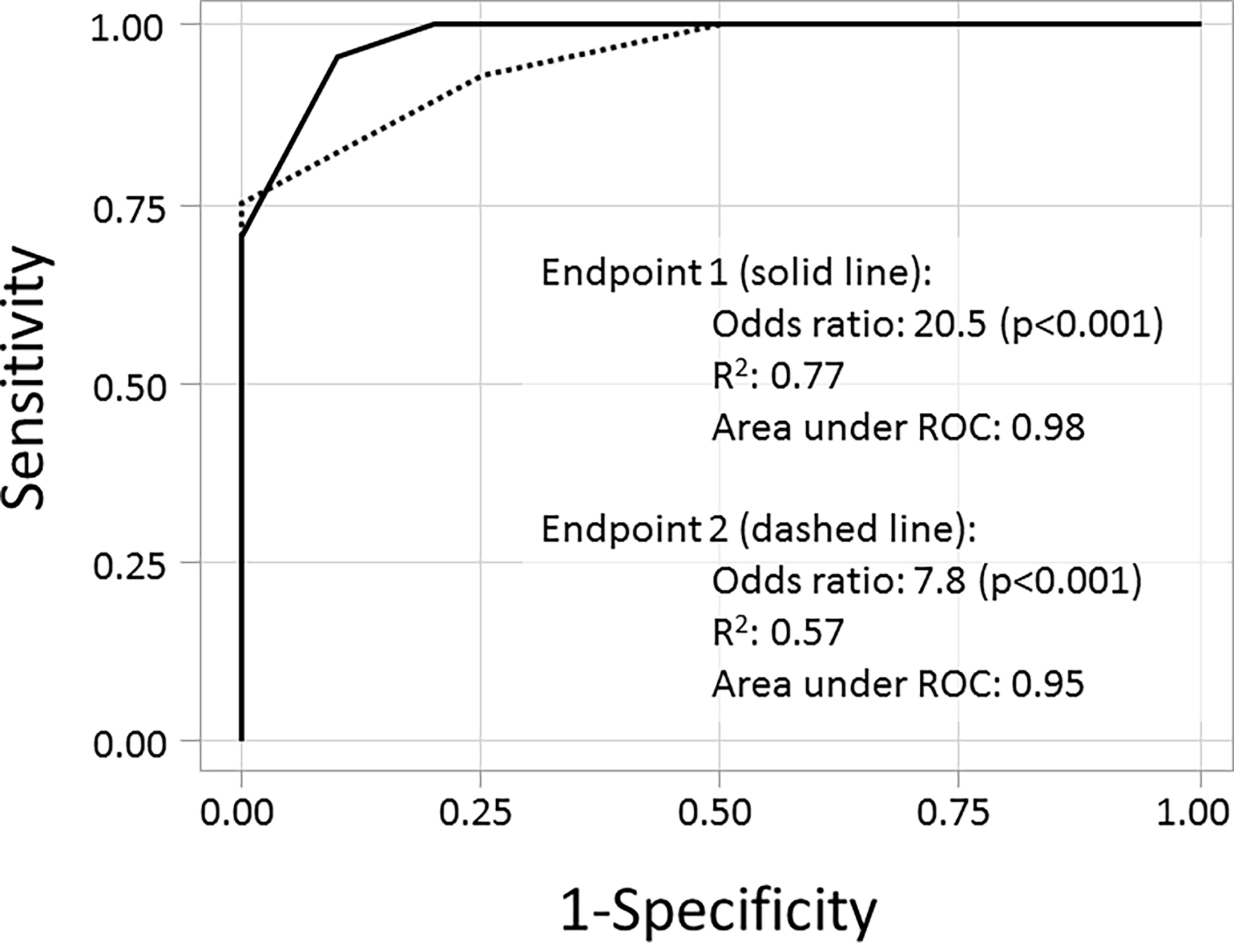
Receiver operator curves using the developed *Shigella* CHIM disease severity score to predict two separate dichotomous endpoints of shigellosis. Footnote: Receiver operator curves using a *Shigella* disease score to predict two separate dichotomous endpoints as follows. Endpoint 1 (solid line): diarrhea (≥2 loose stools ≥200 grams over 48 hours or a single loose stool ≥300 grams) OR fever (oral temperature ≥100.0°F) OR gross blood confirmed by hemoccult in ≥1 loose stool; Endpoint 2 (dashed line): severe diarrhea (≥6 loose stools in 24 hours or >800 grams of loose stool in 24 hours) OR moderate diarrhea (4–5 loose stools in 24 hours or 401–800 grams of loose stool in 24 hours) AND [fever (oral temperature >100.4°F) or with moderate enteric/constitutional symptoms (nausea, vomiting, abdominal cramps/pain, myalgia, arthralgia, rigors, tenesmus, fecal urgency)] OR gross blood confirmed by hemoccult in ≥2 loose stools in 24 hours and enteric/constitutional symptoms (nausea, vomiting, abdominal cramps/pain, myalgia, arthralgia, rigors, tenesmus, fecal urgency).

**Fig 5:**
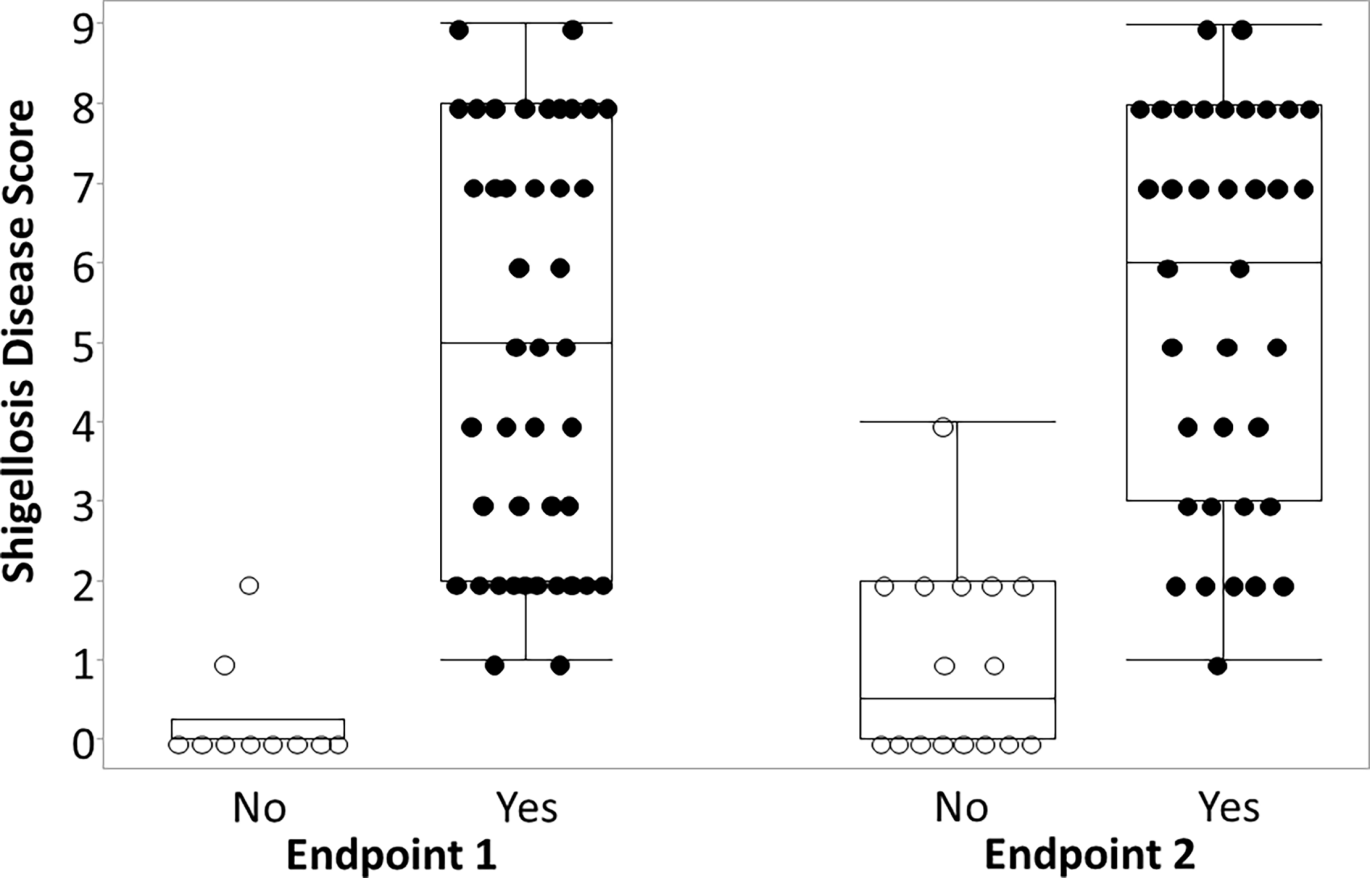
Box and whisker plot of developed *Shigella* CHIM disease severity score by previously utilized dichotomous endpoints for shigellosis. Footnote: Endpoint 1 (solid line): diarrhea (≥2 loose stools ≥200 grams over 48 hours or a single loose stool ≥300 grams) OR fever (oral temperature ≥100.0°F) OR gross blood confirmed by hemoccult in ≥1 loose stool; Endpoint 2 (dashed line): severe diarrhea (≥6 loose stools in 24 hours or >800 grams of loose stool in 24 hours) OR moderate diarrhea (4–5 loose stools in 24 hours or 401–800 grams of loose stool in 24 hours) AND [fever (oral temperature >100.4°F) or with moderate enteric/constitutional symptoms (nausea, vomiting, abdominal cramps/pain, myalgia, arthralgia, rigors, tenesmus, fecal urgency)] OR gross blood confirmed by hemoccult in ≥2 loose stools in 24 hours and enteric/constitutional symptoms (nausea, vomiting, abdominal cramps/pain, myalgia, arthralgia, rigors, tenesmus, fecal urgency).

The *Shigella* disease score was also significantly associated with increasing odds of being characterized as meeting a previously utilized dichotomous endpoint. We applied two previously utilized dichotomous shigellosis endpoints to the abstracted data and assessed the ability of the *Shigella* disease score to predict categorization into those endpoints. As shown in Fig 4, the odds of being categorized as having Endpoint 1 (diarrhea, fever or dysentery) increased by 20.5-fold (p<0.001) for each one-point increase in the disease score yielding an area under the ROC of 0.98. Similarly, the odds of being characterized as having Endpoint 2 (severe diarrhea or moderate diarrhea/dysentery with symptoms) increased by 7.8-fold (p<0.001) for each one point increase in the *Shigella* disease score (area under ROC: 0.95). In addition to significantly predicting dichotomous endpoints, the *Shigella* disease severity score enabled a more robust characterization of post-infection illness characterization than the dichotomous endpoint (Fig 5). Specifically, comparing those that did and did not meet a dichotomous endpoint, for several subjects, there was little difference in the disease severity highlighting the somewhat arbitrary nature of the endpoints utilized to date. Additionally, among those meeting either of the primary ‘shigellosis’ endpoints, severity scores ranged from 1 to 9, indicating a spectrum of illness uncharacterized by the dichotomous endpoints.

## Discussion

Herein we have described the attributes of shigellosis from multiple controlled human infection models and, based on the distribution and co-occurrence of the signs and symptoms of shigellosis post-infection, have proposed a disease severity score for use in *Shigella* CHIM studies. We internally validated the disease score based on its ability to predict previously utilized dichotomous endpoints as well as demonstrated its strong correlation with results from a hierarchical cluster analysis and principal components analysis. Based on these results, the proposed *Shigella* CHIM disease score is an improvement on previously utilized dichotomous endpoints. In particular, the disease score appears to better discriminate clinically consequential signs and symptoms experienced across subjects. As outlined in Fig 5, the use of a dichotomous endpoint is sub-optimal as it does not adequately describe the complex syndrome associated with an invasive enteric disease process. As a result, subjects with very common disease symptoms can be dichotomized in ways that may be less meaningful for vaccine development. Specifically, it is clear that while some subjects may present with a relatively mild illness, others present with full-fledged shigellosis that, if seen in an ambulatory setting, would necessitate additional management modalities to include IV rehydration, antibiotic treatment and clinical observation. Our definition, which further captures the signs and symptoms associated with clinically consequential disease, is more amenable for field settings, and would likely also better predict the bridging of results from the CHIM to the target population and setting. The role of subjective symptoms has the potential to introduce bias; however, application of the Shigella CHIM in a randomized, double-blind, placebo-controlled setting, would minimize the risk of differential misclassification. Additionally, it is important to note that utilizing symptom-based measures as a component of disease severity is also consistent with recent recommendations associated with travelers’ diarrhea management [20].

The most recent applications of the *Shigella* CHIM are focused on assessing the preliminary efficacy of prototype *Shigella* vaccine candidates. These studies are limited in the number of facilities capable of conducting *Shigella* CHIM and the number of beds available at each facility. *A priori* sample size calculations for these types of studies are dependent on two estimates, placebo attack rate and presumed vaccine efficacy and oftentimes these two estimates need to be appropriately balanced. For example, a higher placebo attack rate enables smaller sample sizes to show significant differences in the attack rate between placebo and vaccine groups presuming a constant efficacy estimate. There are two methods of increasing the placebo attack rate. The first option is to modify host susceptibility (by fasting, pretreatment, etc.) or by raising the inoculum dose in such a way to increase the proportion of subjects meeting an endpoint. While this may seem suitable, there is legitimate concern that such modification increases the artificiality of the model potentially overwhelming an ordinarily effective vaccine-induced immune response in protection of a naturally occurring infective encounter. This may also introduce safety concerns. Another option would be to utilize an endpoint that captures a higher proportion of subjects’ disease manifestations. While this may be amenable in certain scenarios, the prototype product must then be able to protect against all spectrum of disease included in that endpoint. To date, most vaccine candidates are targeting moderate-severe shigellosis. Studies considering a broader spectrum of disease must appropriately adjust anticipated efficacy estimates to cover the milder illness included in the more complete disease spectrum.

The *Shigella* disease score may minimize the impact of this equipoise by better quantifying and capturing the full spectrum of disease in the study groups. The use of continuous and ordinal endpoints is more statistically efficient than dichotomous endpoints, requiring smaller sample sizes to differentiate study groups [21]. This may prove to be important not only in assessing the efficacy of a single vaccine candidate, but as an important tool for portfolio management. In particular, the use of the *Shigella* CHIM to assess prototype *Shigella* vaccine candidates in product development pipeline is increasing. Down-selection decisions will need to be made based on the product attributes to include cost of goods, feasibility and studies in the target population; however, early decision making could be bolstered by the use of the *Shigella* disease score that may enable vaccine to vaccine comparisons not currently feasible with limited dichotomous endpoints.

Clearly the disease score proposed herein needs to be externally validated in studies not utilized for its development. In particular, the score was developed using only data from *Shigella* CHIM that utilized *S*. *flexneri* 2a strain 2457T. While that strain is the most commonly utilized in *Shigella* CHIMs, the *S*. *sonnei* strain 53G, which may have a different clinical disease profile that *S*. *flexneri*, is also being re-developed for use in vaccine studies [22]. Applying the disease to those studies would provide external validation of this endpoint and further justify its use in subsequent *Shigella* CHIM. Of particular interest would be the application of the disease score to *Shigella* CHIM assessing prototype vaccine candidates. Similar to the recent development and application of the ETEC CHIM’s severity score [15], the ability of the disease score to differentiate vaccinated and placebo subjects will cement its use in the future as a secondary, or even a primary, endpoint.

## Supporting information

S1 Fig Results of principal component analysis with signs and symptoms from proposed Shigella CHIM disease severity score. Footnote: We performed a principal component analysis (PCA) of the signs and symptoms proposed for inclusion into this Shigella CHIM disease severity score. Variables included were as follows: maximum 24 hour loose stool output frequency and volume, total loose stool output frequency and volume, maximum observed temperature, presence/absence of gross blood in multiple loose stools, severity of: vomiting, nausea, abdominal cramps or pain, malaise, myalgia, arthralgia, headache, anorexia. Fig 1A shows the eigenvalues and the percent of variability described by each component. Fig 1B is a two dimensional graph of the first two components from the PCA.
(TIFF)

S2 Fig Correlation between principal component 1 and proposed Shigella CHIM disease severity score. Footnote: Component 1 from the PCA, accounting for 51.5% of the variability in the distribution of the included signs and symptoms, was strongly associated with Shigella CHIM disease severity score (Spearman rho: 0.97; p<0.0001).
(TIFF)
