# Supplementary figures and images for: Clinical endpoints in the controlled human challenge model for Shigella: A call for standardization and the development of a disease severity score
Source: PLoS One. 2018 Mar 28;13(3):e0194325. doi: 10.1371/journal.pone.0194325 (PMC5874036; doi:10.1371/journal.pone.0194325)

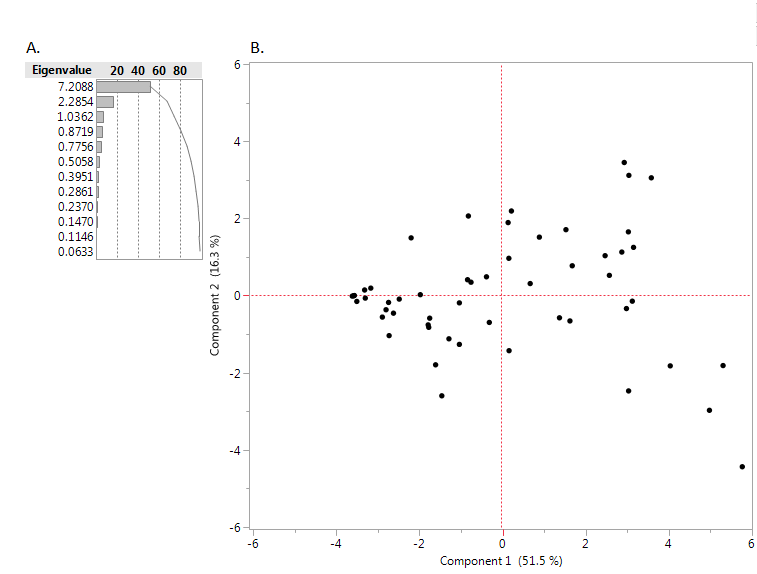

Supplement: S1 Fig — Footnote: We performed a principal component analysis (PCA) of the signs and symptoms proposed for inclusion into this Shigella CHIM disease severity score. Variables included were as follows: maximum 24 hour loose stool output frequency and volume, total loose stool output frequency and volume, maximum observed temperature, presence/absence of gross blood in multiple loose stools, severity of: vomiting, nausea, abdominal cramps or pain, malaise, myalgia, arthralgia, headache, anorexia. Fig 1A shows the eigenvalues and the percent of variability described by each component. Fig 1B is a two dimensional graph of the first two components from the PCA. (TIFF) [file pone.0194325.s001.tiff]

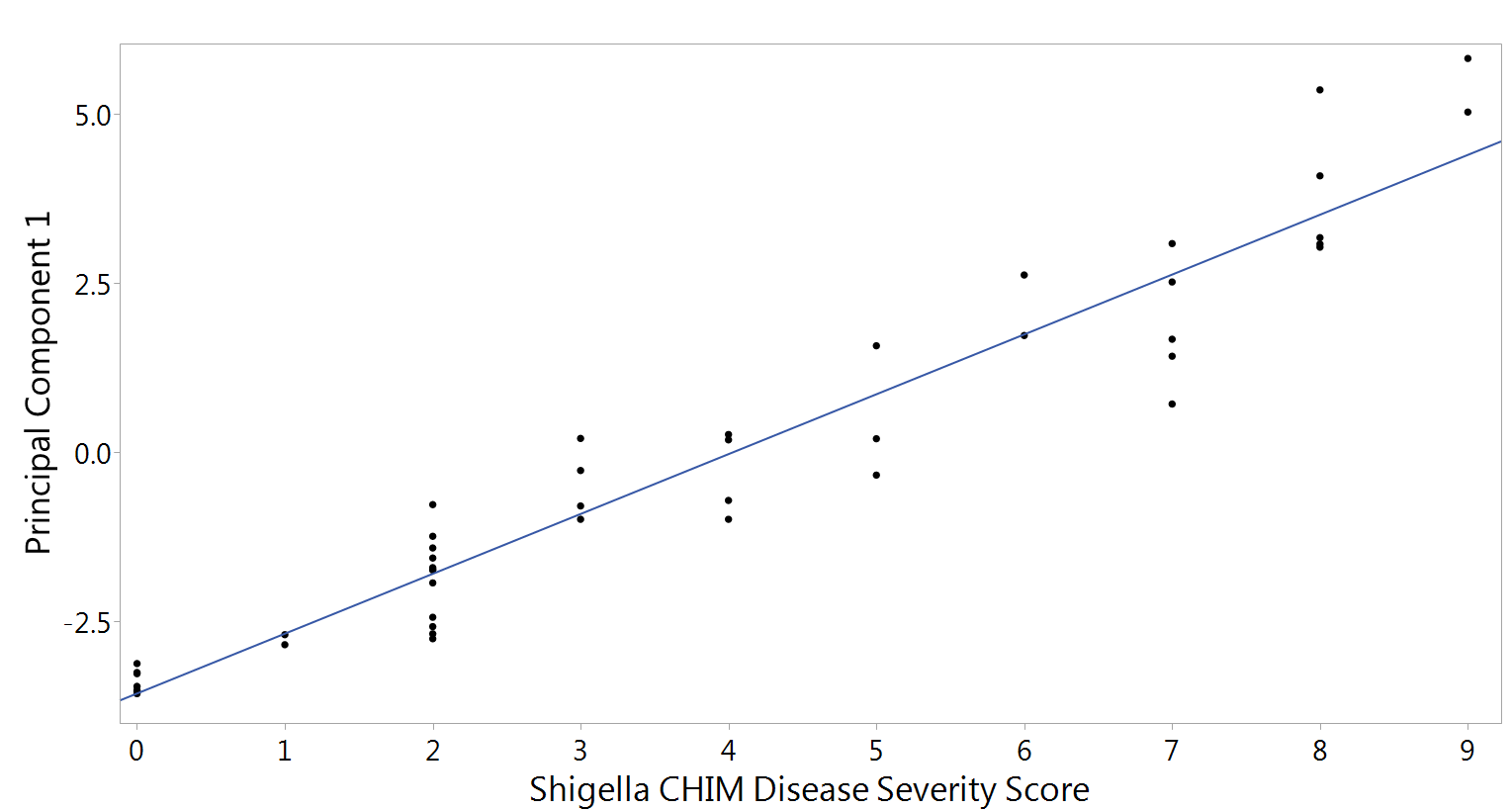

Supplement: S2 Fig — Footnote: Component 1 from the PCA, accounting for 51.5% of the variability in the distribution of the included signs and symptoms, was strongly associated with Shigella CHIM disease severity score (Spearman rho: 0.97; p<0.0001). (TIFF) [file pone.0194325.s002.tiff]
